# Mr. Trye, on the Cow-Pox Inoculation

**Published:** 1804-11-01

**Authors:** Charles Brandon Trye

**Affiliations:** Senior Surgeon to the County Hospital Gloucester


### *To the Editors of the Medical and Physical Journal.*

GENTLEMEN,

WHEN Dr. Jenner first introduced Vaccine Inoculation, I declined adopting it. Inoculation with the small-pox I had long practised without a single loss; I had also fixed opinions in physiology, which militated against what was advanced by himself and his friends. In process of time, however, such a mass of clear, undisputed, decisive evidence came forward in support of the newly-discovered preservative, as to be irresistible to a mind not hardened beyond the susceptibility of conviction; and, consequently, whatever might have been my previous notions, or my habits of thinking, I could no longer persist in the use of variolus matter.

I will not say, that my own practice, in inoculating with cow-pox matter, has been so considerable as that of many others, or that I have made a variety of experiments, with a view to understand or explain any of the phenomena of the disease; but I will say, that in the small-pox, both natural and inoculated, my experience has been ample; and from that experience alone, I *was* enabled to compare the merits of small-pox inoculation with those ascribed to the Jennerian practice. From *my own* experience, then, I can assert, first, that whatever *has been said against the sufficiency of* cow-pock matter, as a security against variolous infection, may be also *said with truth against small-pox matter*, as a similar security. From my own experience I can, secondly, assert, that the subsequent ill effects which *have been said* to follow cow-pox, have, in a ten-fold greater degree, followed small-pox. And lastly, from my own knowledge, I can assert, (and who of long standing in the profession cannot do the same?) that many instances of mortality have happened in small-pox inoculation, whilst amongst *all which has been said,* not a single example appears of death from cow-pox.

In

In behalf of my first assertion, I can recollect numerous facts; but, as I write for the public, and on a most important subject, I will state nothing in support of that assertion, which shall rest solely upon my own credibility or memory; I will, therefore confine myself to the three following cases:—

Mr. John Phillpotts, of this city, well known and esteemed in his profession of the law, was inoculated with the small-pox in his infancy, together with an elder sister, by their father, *with the same* matter, *at the same* time, and both were nursed by the mother, and two persons accustomed to small-pox, of good judgment, and now living. The young lady had the disease to an alarming virulence; the boy's arm inflamed, he was indisposed, and had four or five eruptions on different parts of his body; and Mrs. Phillpotts says, they appeared to her to go on after the manner of other small-pox pustules. In his twenty-first year, I was desired to visit him as being ill with some eruptive fever. He had spots just appearing in different parts of his body; the next time I saw him, nothing but the positive assertion of himself and his friends, that he had had the small-pox, could have made me doubt that they were variolous. On the following day that doubt was entirely removed. He had a plentiful crop of pustules of the distinct kind, which went regularly through their stages of suppuration and scabbing.

In September 1794, I inoculated a daughter of Mr. John Rudhall, of this city, with matter, which *I had taken myself* from a variolous subject. The child's arm inflamed, she was indisposed, and had a few eruptions, which did not suppurate. About twelve months after, I inoculated her again, and she had then the distinct small-pox, with all its usual circumstances.

Mr. Cooke, an eminent apothecary of this city, desired me to see a patient, who had some years before been inoculated by a practitioner of respectability and experience, for the small-pox, together with ten others, in the Gentleman's own Small Pox House. The patient supposed that he then received and went through the disease, and the inoculator assured him of it. When we visited him, he was then blind with small-pox, which went through its usual stages.

In support of my second assertion, I need not stake my own credibility at all. My experience can only coincide with the testimonies already before the public, of the small-pox rousing up scrophula in all its malignant varieties, ties, and being followed by phlegmons, ophthalmias, &c. while nothing beyond cutaneous eruptions has, to the best of my recollection, been imputed to the cow-pox.

But as to my third assertion, its truth is so universally known, that all proof is unnecessary.

I shall go then to the inferences to be drawn from what has been premised. From the cases supporting the first assertion, it appears, first, either that some individuals may receive the small-pox infection twice; or, else, that the patient may be infected to a certain degree with variolous matter, but not so as to make an indelible impression on the constitution. In either case, their inoculation with the small-pox has no advantage, as a protecting security, over the cow-pox. Let it be said, that the practitioner who inoculated the patient supposed to be infected a second time, was, in the first instance, either inattentive or deceived by doubtful appearances; or that the first time his patient was not inoculated with real small-pox matter, or with small-pox matter in a proper state. To the first supposition, it must be answered, that in the general practice of cow-pox inoculatoin, it is not to be believed that operators will be more sagacious, more discriminating, or more attentive than their predecessors have been in small-pox inoculation; and to the second, that similar errors are just as likely to prevail in vaccine inoculation: So that the conclusion must be, either that there are individuals, in whom the susceptibility of the small-pox is not destroyed by a well-conducted process either of the cow-pox or small-pox inoculation; or that, in the instances, when either the one or the other failed to secure the individual against future small-pox, the process did not go so far as to make the proper impression on the constitution; or lastly, that in the inoculation, improper matter must have been used; *which, however, could not have been the case in the two first examples given above, in proving my first assertion.*

Three instances have been brought forward, amidst the voluminous writings for and against the cow-pox inoculation, where it failed of securing the patient against small-pox; two by Mr. Goldstone, of Portsmouth, and one in the London Papers of the beginning of this month. Whether the patients were inoculated with genuine cow-pox matter or not, I will not inquire; I will admit their weakening our confidence in vaccination to a certain degree. But these three failures, amid the collected experience of the Profession in general, are here met by the experience experience of a single individual, in a provincial town, with an equal number of cases, equally weakening our confidence in small-pox inoculation. In this repect, then, let the two inoculations be supposed to stand upon equal grounds. But let the *consequences* of one be weighed against those of the other, and the scale of vaccination must incalculably preponderate. In immediate danger to the individual, in remote mischief to his constitution, the cow-pox has infinitely the advantage. To this let us add, that while with *the cow-pox* the practitioner, at the worst, injures no one except his patient, *with the small-pox* he may deal misery and destruction among his neighbours, far beyond the limits of his operating; that in the one he is continually risking the dissemination of a loathsome and mortal disease, while in *the other* he is conducing to the extermination of that pestilence from among mankind. Let us, then, turn to common sense, and ask her, which she would prefer?

I am, &c.

*Gloucester, Oct.* 6, 1804. CHARLES BRANDON TRYE,
Senior Surgeon to the County Hospital

